# How to approach the study of syndromes in macroevolution and ecology

**DOI:** 10.1002/ece3.8583

**Published:** 2022-03-14

**Authors:** Miranda A. Sinnott‐Armstrong, Rocio Deanna, Chelsea Pretz, Sukuan Liu, Jesse C. Harris, Amy Dunbar‐Wallis, Stacey D. Smith, Lucas C. Wheeler

**Affiliations:** ^1^ Department of Ecology and Evolutionary Biology University of Colorado‐Boulder Boulder Colorado USA; ^2^ Department of Chemistry University of Cambridge Cambridge UK; ^3^ Instituto Multidisciplinario de Biología Vegetal IMBIV (CONICET‐UNC) Córdoba Argentina; ^4^ Departamento de Ciencias Farmacéuticas Facultad de Ciencias Químicas (FCQ, UNC) Córdoba Argentina

**Keywords:** convergent evolution, pollination syndromes, syndromes, trait evolution

## Abstract

Syndromes, wherein multiple traits evolve convergently in response to a shared selective driver, form a central concept in ecology and evolution. Recent work has questioned the existence of some classic syndromes, such as pollination and seed dispersal syndromes. Here, we discuss some of the major issues that have afflicted research into syndromes in macroevolution and ecology. First, correlated evolution of traits and hypothesized selective drivers is often relied on as the only evidence for adaptation of those traits to those hypothesized drivers, without supporting evidence. Second, the selective driver is often inferred from a combination of traits without explicit testing. Third, researchers often measure traits that are easy for humans to observe rather than measuring traits that are suited to testing the hypothesis of adaptation. Finally, species are often chosen for study because of their striking phenotypes, which leads to the illusion of syndromes and divergence. We argue that these issues can be avoided by combining studies of trait variation across entire clades or communities with explicit tests of adaptive hypotheses and that taking this approach will lead to a better understanding of syndrome‐like evolution and its drivers.

## Graphical abstract

Syndromes, wherein multiple traits evolve convergently in response to a shared selective driver, form a central concept in ecology and evolution. Recent work has questioned the utility and indeed the existence of some of the classic syndromes, such as pollination and seed dispersal syndromes. Here, we discuss some of the major issues that have plagued research into syndromes in macroevolution and suggest an approach to avoid these issues.

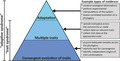

## 1 INTRODUCTION

One of the most striking and commonly studied phenomena in biology is that of convergent evolution, whereby distantly related species evolve similar phenotypes as adaptations to similar selective pressures (Darwin, 1859; Ollerton et al., 2009; Waser et al., 2011). When this convergence involves multiple traits, it is often called a “syndrome.” Historically, the term “syndrome” has been applied in a wide variety of contexts. For instance, “syndrome” has been used to describe cases where traits are fixed in a population or species (e.g., the repeated loss of eyes and pigmentation in cave fish; Strecker et al., 2012), polymorphic (such as behavioral syndromes or personalities; Sih et al., 2004), or plastic (e.g., plant defense syndromes; Agrawal & Fishbein, 2006). Syndromes have also been described that are restricted to a single lineage or a small number of lineages (tree lobsters; Buckley et al., 2009) and to cases where similar combinations of traits have evolved many times across diverse clades of the tree of life (e.g., flight; Dudley, 2002; Rayner, 1988). Further adding to the confusion, while the term “syndrome” is popular in the botanical literature (e.g., pollination syndromes, dispersal syndromes, succulent syndromes; Janson, 1983; Ogburn & Edwards, 2009; Waser et al., 1996), alternative terms are often used by zoologists to describe the same phenomenon of repeated evolution of multiple traits (such as “ecomorphs” in *Anolis* lizards; Beuttell & Losos, 1999). Finally, the concept of a “syndrome” also overlaps with other ideas in ecology and evolution, including “strategy,” “specialization,” and even “system” (Agrawal, 2020; Tripp & Manos, 2008). This plethora of uses of “syndrome” and related terms has caused considerable confusion about what a syndrome is and what it is not, and how to study the evolution and ecology of multiple convergent traits.

Historically, “syndromes” have been in two primary arenas. In macroevolution, syndromes are typically tested by examining correlated evolution of traits along a phylogeny. In community ecology, species are often classified into different syndromes based on their trait combinations. In both arenas, the “syndrome” of traits is typically assumed to result from adaptation to a single selective driver (e.g., primary pollinator, preferred habitat). In some cases, adaptation to a single selective driver does seem likely, such as the repeated changes in phenotype following transitions to lake or stream habitats in sticklebacks (De Lisle & Bolnick, 2020; Thompson et al., 2017). However, in other cases, there are likely to be multiple and/or competing selective drivers, such as in pollination syndromes where most species are visited by multiple animal species although a “primary” pollinator is usually assumed (Rosas‐Guerrero et al., 2014; Sahli & Conner, 2011; Sun et al., 2014). Identifying cases where a single selective driver is likely versus those where there may be multiple or competing drivers can be very difficult, yet is essential to understanding whether a particular combination of traits evolves repeatedly under the same conditions.

Building a broad and balanced understanding of the role of selective agents in driving convergent, multitrait evolution is critical to understanding the link between adaptation and trait evolution. However, researchers often assume the existence of syndromes, rather than explicitly test whether their system exhibits the requisite features. The presupposition of syndromes causes a number of problems. First, it leads researchers to measure species with traits prototypical of the proposed syndrome while ignoring species with intermediate traits as outliers, generalists, or intermediates. In fact, continuous variation in traits is common even in systems that have traditionally been interpreted as evolving discrete syndromes, including flowers (Ollerton et al., 2009), fleshy fruits (Janson, 1983; Sinnott‐Armstrong et al., 2018), dry fruits (Wojewódzka et al., 2019), dispersal traits of terrestrial animals (Stevens et al., 2014), migration‐associated traits in birds (Piersma et al., 2005), and body morphology in sea snakes (Sanders et al., 2013), among others. Such intermediate species are often dismissed as outliers, rather than treated as what they are: valuable information about the drivers of evolution.

Second, the presupposition of syndromes leads to the telling of “just‐so” stories about evolution (Gould & Lewontin, 1979; Olson & Arroyo‐Santos, 2015). One example occurs in “anachronistic fruits,” where very large, fleshy fruits with a protective husk or rind are thought to be adapted to dispersal by extinct megafauna such as elephants, giant lemurs, or extinct gomphotheres (Albert‐Daviaud et al., 2020; Guimarães et al., 2008; Janzen & Martin, 1982). The osage orange (*Maclura pomifera*) is often held up as a prototypical example of this phenomenon, whose persistence, despite the extinction of putative dispersers more than 10,000 years ago (Guimarães et al., 2008), has been extended by humans (Smith & Perino, 1981). Despite the neatness of the story that these large fruits were consumed by now‐extinct large animals, the idea is controversial for a number of reasons. Empirical tests of whether ingestion by modern‐day analogs of extinct elephants and horses increases seed germination rate suggest that *M*. *pomifera* seeds do not survive processing in modern horse intestines, and passage through elephant guts decreased rate of germination (Boone et al., 2015). The seeds of another “anachronistic” fruit, *Diospyros virginiana*, survived and germinated following passage through the gut of native, small dispersers such as racoons and coyotes (Rebein et al., 2017). Furthermore, fruit and seed size can evolve rapidly following the extinction of large dispersers (Galetti et al., 2013), yet *M*. *pomifera* fruits have remained large for thousands of years. The confusing evidence in this particular example should lead researchers to wonder what other factors may influence the evolution of such unusual fruits, yet instead researchers tend to favor these “just‐so” stories. This is the same difficulty that occurs in studies of adaptation, but is likely exacerbated by the fact that multiple traits are involved.

Much of the allure of the concept of syndromes comes from the desire to infer adaptation from easily observed traits, especially when the work of experimentally testing the evolutionary driver is challenging, as it usually is. Because of their potential power in making such inferences, syndromes are of particular interest for a number of reasons. Perhaps most notably, paleobiology largely relies on the inference of adaptation and ecological function from morphological traits preserved in the fossil record (e.g., Deanna et al., 2020; Hörnschemeyer et al., 2010; Pritchard et al., 2021): without reliable understanding of the connection between traits and adaptation, accurately interpreting the ecology of the fossil record is impossible. This is true both when examining individual fossils (e.g., Pritchard et al., 2021) as well as when incorporating both extant and extinct taxa on the same tree in studies of morphological evolution (e.g., Federman et al., 2016). Syndromes are also used in a variety of other contexts, such as to predict responses to abiotic changes or to predict species interactions based on morphology and other traits (Dehling et al., 2016; Ficetola et al., 2016; Michel et al., 2017; Phillips & Shine, 2006; Schleuning et al., 2020).

These uses of syndromes (and traits more broadly) have real implications for conservation and for our ability to accurately predict responses of species to climate change, both in terms of species' abilities to migrate or evolve in new environmental conditions, as well as their ability to maintain vital species interactions. The validity of results based on syndromes depends largely on the degree to which those syndromes can actually be used to predict ecology (e.g., Thompson et al., 2017). Unfortunately, both evolutionary biologists and ecologists have tended to focus on only one side of the syndromes adaptation question. Evolutionary biologists generally focus on patterns of convergence and correlated evolution between traits and hypothesized evolutionary drivers, with only coarse understanding of the ecology of those traits. Ecologists tend to focus on classifying species within a community into categories based on their trait combinations, while limited emphasis on the evolutionary history of those traits and species.

Here, we describe an approach to studying syndromes that addresses these challenges and encourages more rigorous, integrative research into multitrait convergence. First, we define “syndrome” as it has been historically used in the literature and suggest a differentiation into subcategories of “trait syndromes” (where an observation of convergence has occurred, without tests of adaptation) and “adaptive syndromes” (where such traits can reliably infer adaptation to a particular driver). Then, we explore a hypothetical example to illustrate common approaches to the study of syndromes and their potential pitfalls. We identify a number of problems with the way that researchers typically approach the study of syndromes, including the assumption that correlated evolution is strong evidence of adaptation, sampling bias that creates the illusion of syndromes, and the study of traits that are not especially relevant to the hypothesized evolutionary driver. Finally, we propose an approach to studying syndromes that overcomes these problems and allows us to build evidence‐based evolutionary narratives about syndromes of traits and adaptation.

### 1.1 What is a syndrome?

**FIGURE 1:**
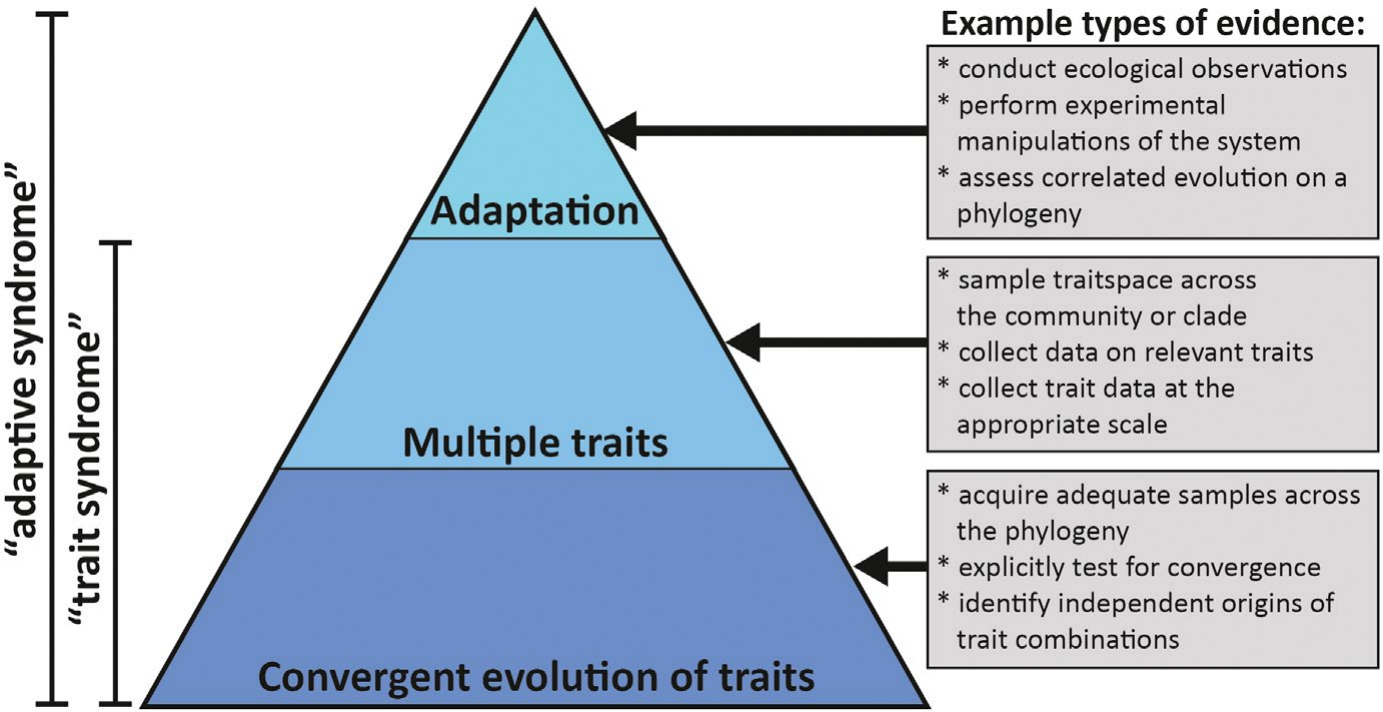
Schematic illustration of the three main features of syndromes (adaptation, multiple correlated traits, and convergent evolution of traits) as well as a sample of approaches for demonstrating each of those features within a study system

The diversity of scenarios in which the term “syndrome” has been applied, as well as the variety of other terms with partly or completely overlapping meanings, means that the definition of a “syndrome” varies considerably across studies. Broadly speaking, the literature contains two major kinds of syndromes: syndromes which have been described based solely on convergent evolution of a combination of traits with no tests of adaptation (e.g., Sinnott‐Armstrong et al., 2020) and syndromes in which adaptation to a particular driver has been tested (e.g., Sanders et al., 2013). Here, we differentiate these two kinds of syndromes into “trait syndromes” and “adaptive syndromes.” Based on a review of the literature, we identify three criteria which are usually used to characterize syndromes across systems, scales, and clades: (1) convergent evolution of traits; (2) involvement of multiple traits; and (3) adaptation of those traits to a selective driver (Box 1; Figure 1). We define “trait syndromes” as cases where convergent evolution of multiple traits has been demonstrated (criteria 1 and 2), but the link to adaptation has not yet been established. “Adaptive syndromes,” on the other hand, have convergent evolution of multiple traits, but also have evidence of adaptation to a selective driver and may even be shown to be predictive of that evolutionary driver (criteria 1, 2, and 3; Box 1). Clustering of traits in trait space is sometimes also considered important (Ollerton et al., 2009), but here we consider clustering of traits to be one possible line of evidence of adaptation, rather than a necessary feature of the distribution of trait values in a syndrome.

To demonstrate each of these criteria, multiple lines of evidence are generally needed (Figure 1). For instance, convergent evolution of traits requires adequate phylogenetic sampling, as well as consideration of the degree to which ancestral states and independent origins can be identified given the tree and trait histories. Ancestral state reconstructions are hypotheses of evolutionary history, but come with a variety of caveats and uncertainties and as such must be treated with caution (Holland et al., 2020; Reyes et al., 2018; Wheeler et al., 2016). For multiple traits, pleiotropy and other types of genetic linkage can cause the appearance of multiple unrelated traits evolving in correlation (Funk et al., 2021; Raia et al., 2010). Testing for such genetic linkages may not always be simple, but other lines of evidence can provide evidence that multiple traits evolve independently, such as imperfect correlation of traits (e.g., species which have only two of the three traits involved in a syndrome). For adaptation, correlation between traits and putative evolutionary drivers on a phylogeny may suggest adaptation (see discussion below), but other measures such as ecological studies or experimental manipulations provide critical data toward a hypothesis of adaptation beyond the use of correlated evolution alone. Consideration of the strength of evidence from both an evolutionary and an ecological perspective will greatly enhance confidence in the inference of relationships between trait syndromes and adaptive drivers, as well as the predictive value of syndromes.

### 1.2 Setting the stage: A hypothetical case study

Let us travel, for a moment, to a hypothetical archipelago. On the first island, we find a small, iridescent beetle, and a large, black beetle. The small iridescent beetle runs along branches and munches on leaves, while the large black beetle buries itself in the leaf litter and eats small insects. On the next island, we find a similar pair of beetle species—small and iridescent, large, and black. We observe these beetles on each island for several months and begin to suspect that the iridescence provides camouflage in variable light environments such as occur on exposed branches (Kjernsmo et al., 2020). Black color may provide camouflage against the leaf litter, enabling the large beetle to forage on the ground undetected. After wrapping up fieldwork, we write it up: new beetle syndromes on islands, possibly adapted to foraging style!

Syndromes are commonly discovered and first described in this fashion, with the observation of covarying traits across species and hypotheses of potential drivers. Often, a body of literature is built on the assumption of an adaptive connection. However, there are problems with this approach, which can give rise to the illusion of a far more complete understanding of the natural system than has actually been achieved (Ollerton, 2021). As described above, adaptive syndromes have three primary features: convergent evolution, multiple traits, and adaptation to an evolutionary driver (Figure 1). In this hypothetical example, we have not explicitly tested for any of these features. First, we have not tested for convergence of the traits we observed. Without knowing the relationships between species, we cannot say that convergence, rather than inheritance, has occurred. Second, we have not established that changes in the traits are correlated in a phylogenetic context, in part because we have not sampled species beyond the few pairs that were the focus of our hypothetical study. Third, we did not test the association between traits and potential drivers. Simply observing a set of traits that cluster in trait space is not evidence of adaptation to a particular driver without additional data. Below, we describe four fundamental problems with the sloppy approach to describing new syndromes that has occurred in our hypothetical example.

BOX 1 **Definitions of terms used**

Language matters, and in the case of syndromes, the language used to refer to those syndromes matters. We believe that a substantial portion of the confusion in the literature derives from imprecise language. In particular, it is common to name a proposed syndrome after its putative driver, long before such a driver has been critically examined. Pollination syndromes are one prototypical example: even studies that do not find support for pollination syndromes include statements such as “pollination syndrome was a poor predictor of [floral] visitors” (Hilpman & Busch, 2021) simply because this is the language used historically to refer to that syndrome. This results in confusing statements that appear to support the idea of a pollination syndrome when the data suggest otherwise, further obscuring the concept of a syndrome and the strength of evidence supporting (or refuting) that syndrome.

***“Adaptive syndrome”***. Here, we define an adaptive syndrome as having three features: (1) convergent evolution of (2) multiple traits (3) adapted to a particular driver. Typically, different states of the driver—such as different pollinators or different habitats—will result in distinct clusters of traits, although discrete clustering is not necessary. We include convergent evolution as a criterion because the repeated appearance of the trait combination is key evidence for the generality of the association. A collection of traits selected simultaneously in a single lineage serves as a compelling story of the complexity of adaptation, but does not provide the predictability that makes syndromes a valuable concept in the literature.

“***Trait syndrome”***. We propose the term “trait syndrome” to refer to cases where two of the three criteria have been met, namely that there has been (1) convergent evolution of (2) multiple traits. However, in trait syndromes, the adaptive driver remains a hypothesis rather than well‐demonstrated. We propose this terminology in order to differentiate scenarios in which only traits have been studied from those scenarios in which adaptation has also been studied.

By differentiating “trait syndromes” (combinations of traits) from “syndromes” (where the link to selection has been demonstrated), we aim to provide linguistic tools to better highlight cases where adaptation has been well‐studied. We urge researchers to consider the strength of their evidence that their syndrome of study is adaptive to the proposed driver and to name their syndromes (if they choose to do so) with trait‐centric terminology (e.g., “red flower syndrome” rather than “hummingbird syndrome”) until they have sufficient evidence to support the adaptive connection.

### 1.3 Problem 1: Assumption that correlated traits are strong evidence of adaptation

One of the major problems in our hypothetical example is that we did not test explicitly for convergence of traits on a phylogeny. Because traits may be shared due to common ancestry rather than adaptation (Felsenstein, 1985), failing to test for convergence can give the impression of a strong relationship between traits and adaptation that are not a result of selection acting on independent lineages (see Hilpman & Busch, 2021 for a discussion of this in the context of pollination syndromes). For instance, we might travel to a third island and observe another pair of beetles. We might assume that, because they share a similar morphology, they also have the same foraging styles as our original species pairs. However, without knowledge of whether these traits were inherited or independently evolved, the strength of support for an adaptive explanation is low. In building an adaptive story from limited observations of a small number of species pairs, we may simply be testing whether these species are different (Garland & Adolph, 1994), not whether they are adapted to the hypothesized driver. Although it is not impossible to assess adaptive hypotheses for trait combinations that have arisen only a single time, phylogenies large enough to capture multiple origins of a trait syndrome will have much greater power to move toward assessing causal relationships (Maddison & Fitzjohn, 2015; Uyeda et al., 2018).

### 1.4 Problem 2: Inference of adaptation, without testing, from a combination of traits

Making a leap from trait covariation to adaptive explanation may seem farfetched, but it is common practice. Many syndrome studies rely on data about adaptation that is a mix of peer‐reviewed papers, personal observations, and inferred evolutionary drivers based on an organism's traits. For instance, it is common to observe a new flower, examine its color and size, and infer its primary pollinator despite no field observations—and this approach occurs in other systems as well (see, e.g., Bruneau, 1997; Goolsby, 2017; Hingston & McQuillan, 2000 whether a trait syndrome can predict its; Lomáscolo et al., 2008; Valenta et al., 2018; Whittall & Hodges, 2007). These inferred evolutionary drivers are then often used for downstream analyses that test for correlations between traits and adaptive drivers, which is circular reasoning obscured by scientific methods. The more iterations of this type of unsubstantiated inference, the more difficult it becomes for downstream users of the information to determine the quality of evidence supporting an adaptive syndrome. Recent examples illustrate how some phenomena, long believed to be true, were based largely on limited observations that turn out to be incorrect or incomplete when examined more comprehensively (e.g., migratory syndrome in birds, Piersma et al., 2005; territoriality in lizards, Kamath, 2017). Personal observations contribute vital information to evolution and ecology, but should be treated as observations, not generalizable principles.

Demonstrating adaptation is often challenging and time‐consuming and requires different tools depending on the system. In our view, explicit tests of adaptation are crucial to demonstrating adaptive syndromes (Figure 1). From an ecological perspective, field observations and measurements, in addition to manipulations of relevant ecological variables, provide data pertaining to an adaptive link between traits and the hypothesized evolutionary driver (e.g., da Silva & Batalha, 2011). From an evolutionary perspective, finding clustering in trait space that corresponds with the putative selective driver (Agrawal, 2020; Ollerton et al., 2009) is one line of evidence. Identifying correlated evolution of traits and an adaptive driver (if the trait syndrome itself is not used to infer the driver) is another line of evidence (Pagel, 1994, 1999), but should not be used as the sole line of evidence (as described above).

### 1.5 Problem 3: Measuring irrelevant traits

A third major problem with the study of syndromes, as demonstrated in our hypothetical example, is that we did not consider which traits to measure in order to test our hypothesis about the association between traits (size and color) and driver (habitat). Instead, we chose the traits that were obviously different to us, as humans. Choosing obviously different traits is an excellent way to identify interesting differences and may allow us to identify trait syndromes across species. However, such obvious traits may not be appropriate to use to test for adaptation to a particular driver.

In our hypothetical example, let us say that we have examined the beetle community on our islands and observed that beetle size, but not color, is correlated with habitat (perhaps we have since found iridescent beetles in the leaf litter and black beetles in variable light environments). We spend several years observing these beetles in their natural habitats and eventually notice that beetles foraging on branches (which we once thought were camouflaged with iridescent coloring) have a peculiar behavior: They tap the branch several times before scurrying out to munch on leaves, upside down. This new observation leads us to consider other traits that might be adapted to this foraging lifestyle. We find that foot morphology, behavior, and body size reliably differentiate between branch‐foraging and litter‐foraging beetles. In fact, these other traits are highly predictive of foraging style across beetles displaying a variety of colors.

What happened in this extension of our hypothetical example? The obvious trait—coloration—is easily measured. But, the important traits are more difficult to quantify, requiring behavioral assays and microscopic analyses of foot morphology. This may seem obvious from the safety of our office chairs, but measuring the relevant traits, at the appropriate scale, is critical to evaluating adaptation. A classic example of this occurs in ultraviolet (UV) nectar guides in flowers: Humans cannot see UV light, but bees can. Until the scientific community could measure UV reflectance, UV nectar guides remained unknown, yet are central to the ability of bees to find and pollinate flowers (Hansen et al., 2012). As another example, nectar viscosity (which is partly a function of its sugar content) affects the rate at which nectar can be sucked up by pollinators and consequently has a strong influence on the rate at which energy is acquired from that nectar (Pattrick et al., 2020). However, nectar viscosity is measured less often than other floral traits (Parachnowitsch et al., 2019). In both of these examples, assessing adaptation requires measuring the relevant traits, rather than simply traits that are easy for humans to observe and measure.

Methods for identifying the appropriate traits and biological scale to study are not always obvious (Agrawal, 2017, 2020). We encourage a first principles approach to identifying relevant traits: rather than quantifying everything in order to choose the traits most statistically associated with the adaptive driver, it is preferable to develop a hypothesis about the effect of the adaptive driver on trait evolution and then choose traits expected to vary based on that hypothesis. Quantifying a variety of traits is an important way to discover new correlations and hypotheses, but should be treated as a step in the iterative process of studying adaptation rather than the end point (Olson & Arroyo‐Santos, 2015). Reliance on quantifying many different traits in order to see which ones produce statistically significant results leads to problems where studies of the same syndrome all use different traits, which raises the possibility that those traits were cherry‐picked to support the syndrome hypothesis. This kind of variability in traits studied has occurred in many different syndromes, including pollination syndromes (Ollerton et al., 2009), seed dispersal syndromes (Valenta & Nevo, 2020), pace‐of‐life syndromes (Royauté et al., 2018), island syndromes (Juette et al., 2020; Raia et al., 2010), and others. Consequently, while quantifying a diversity of traits may be helpful for exploration, when analyzing adaptive syndromes, it is preferable to choose traits to study that relate directly to the hypothesis of adaptation. Preliminary studies, using for example samples from museum collections or small‐scale trait measurements in a population, could assist researchers in developing and testing their hypotheses and in deciding which traits are relevant to measure for full‐scale studies.

### 1.6 Problem 4: Sampling bias can create the illusion of discrete clusters of traits

A fourth major issue in our example is that our dataset is biased to detect differences, rather than to assess the hypothesis of adaptation in a community and evolutionary context. In our example, our hypothesis was derived from observing two very different species, but we did not know whether those divergent phenotypes represented distinct optima or whether our species occurred along a continuum of variation. For instance, we did not assess whether there are black and iridescent beetles in other habitats, which would cast doubt on the idea that color is associated with habitat. Broad phylogenetic sampling, including of species that do not appear to exhibit the hypothesized syndromes, can reveal patterns in evolution that shed light on the adaptation of a trait syndrome to an adaptive driver. For example, *Anolis* lizard ecomorphs, associated with specific ecological niches, have evolved multiple times, but primarily on islands—continental *Anolis* species differ in morphology from island species (Pinto et al., 2008). By studying the evolution of *Anolis* morphology on a phylogeny, the multiple origins are identifiable and the differing patterns on islands vs. mainland become clear. In particular, the clear clusters of traits in island *Anolis*, vs the wider distribution of traits in continental *Anolis*, provides support for the notion that island evolution is likely the result of adaptation to a particular habitat.

Studies of syndromes in their ecological context can also shed light on whether a given combination of traits is adaptive to a hypothesized evolutionary driver. By sampling entire communities, we can identify traits that covary across the whole community and the degree to which those trait combinations are associated with putative selective drivers. In flowers, pollination syndromes are often thought of as discrete, multivariate optima, but more complete sampling reveals that floral traits rarely match up exactly with the platonic ideal of discrete optima and that there are many intermediate species (Ollerton et al., 2009; Smith et al., 2008, 2009; Tripp & Manos, 2008). This continuum of traits in pollination syndromes suggests that multiple and/or competing drivers may influence flower evolution, which is not accounted for under the simple model of syndromes as adaptation to a primary pollinator. It is important to note that even in a community context, trait variation is shaped by evolutionary history, which should be incorporated accordingly (Cavender‐Bares et al., 2009; Webb et al., 2002).

### 1.7 Linking patterns in traits to adaptation

In studying syndromes, we recommend to carefully test the features of syndromes described here, including (1) convergent evolution, of (2) multiple traits, which (3) reflect adaptation. When testing for these features of syndromes, we urge readers not to rely solely on correlated evolution along a phylogeny as evidence of adaptation, but rather to assemble evidence from a variety of perspectives (such as experimental tests, field observations, genetics, comparative analyses, clustering in trait space, and others).

Demonstrating whether a trait syndrome can predict its putative driver is an additional piece of evidence pertaining to adaptation and is especially important if the hypothesized adaptive syndrome is intended to be used to infer abiotic environment, species interactions, and other ecological factors. For example, observing that carnivorous plants only occur in low‐nutrient environments is evidence that carnivory may be especially adapted to those low‐nutrient environments (Ellison & Adamec, 2018). In our hypothetical beetle example, if we observe that the small and iridescent beetles always occur in the same habitat, and never occur in other habitats, that is evidence of adaptation of that phenotype to that habitat. Confirming, this, however, requires seeking data to test the hypothesis of exclusive occurrence of that phenotype in that habitat—by examining other habitats and species as well. Careful observational or experimental studies will allow researchers to uncover nonexclusive, quantitative, relationships between clusters of traits and the environment. For example, it is common to observe that certain phenotypes substantially skew the distribution of an organism across various environments (e.g., Farkas et al., 2013).

Careful sampling—across phylogenetic and community diversity—provides the larger context needed to avoid biasing toward the detection of syndromes through examination of species with extreme traits and/or traits that appear convergent while ignoring “intermediate” or “generalist” species. While any observation of a natural phenomenon is likely to start with observing traits that humans are easily able to identify and measure, we encourage readers to iterate over their initial observations to develop hypotheses as to the evolutionary drivers of their syndrome of study and then to consider which traits are relevant to that driver (Olson & Arroyo‐Santos, 2015). Finally, we also propose that researchers take care to use the language of traits (e.g., “floral syndromes”) to describe observed clusters of traits before using the language of adaptation (e.g., “pollination syndromes”) until an adaptive link has been demonstrated and tested.

It is equally important to recognize that patterns of trait variation are shaped by many factors beyond adaptation, which can complicate, but also enrich, the study of syndromes. For example, historical contingency is well known to shape adaptive trajectories and has a strong effect on phenotypic outcomes at various scales (Blount et al., 2008; Harms & Thornton, 2014; McGlothlin et al., 2016). These historical effects can also limit the trait space that is accessible to a lineage and result in incomplete convergence (Grossnickle et al., 2020; McCurry et al., 2017). Even when convergence is complete, the coordinated evolution of multiple traits can occur without coordinated selection on those traits. For example, traits like flower length and width are genetically correlated, which could explain, at least in part, their coordinated evolution at the macroevolutionary scale (Wessinger & Hileman, 2016). Similarly, a chromosomal inversion in *Acanthis* songbirds results in genomic linkage between beak shape and bird color, which creates the appearance of simultaneous selection on both traits even though the correlation is a result of genetic architecture (Funk et al., 2021). Dissecting the interplay of genetic architecture and the multitrait response to selection has a long history in quantitative genetics (Cheverud, 1984; Lande, 1979; Saltz et al., 2017) and merits greater integration with the study of classic trait and adaptive syndromes.

## 2 CONCLUSIONS

Despite widespread interest in syndromes among evolutionary biologists, their study has been haphazard, unsystematic, and rife with circularity. Here, we outline four major problems with the ways that syndromes have been treated in evolutionary biology. (1) Trait syndromes have been used as evidence of adaptation, but should be considered hypotheses that must be tested. (2) Adaptation is inferred from traits without sufficient testing. (3) Easy to measure traits, which may not be relevant to the hypothesis of adaptation, are often used. And finally, (4) syndromes are often identified based on biased samples of the most morphologically divergent species. Together, these issues have meant that syndromes of traits are regularly described and attributed to an adaptive driver with little evidence actually linking the two.

The study of syndromes is important because syndromes are regularly used to infer ecology in a variety of contexts, including fossil organisms and newly discovered species, as well as to predict responses to abiotic change and/or species interactions. Through studying syndromes more rigorously, we improve our ability to conduct studies that rely on adaptive syndromes and have greater confidence in our inferences and predictions. Furthermore, many questions about convergence and adaptation become accessible. For example, what is the evolutionary trajectory of different syndromes—do traits evolve in the same order, or in different orders, as a syndrome is assembled? To what extent do pleiotropy and other genetic linkage mechanisms explain the observation of syndromes? To what extent are syndrome traits convergent across biological scales (e.g., genetic, protein, and phenotypic) and to what extent do species evolve unique adaptations? Some theoretical questions about syndromes also remain. For instance, are there synergistic interactions between individual traits of a syndrome, such that their combined contributions are greater than the sum of its parts? As traits related to a syndrome accumulate in a lineage, do they offer diminishing returns, such that a subset of traits is sufficient for adaptation to the selective driver? How does the relative size of fitness contributions from individual syndrome traits affect evolutionary trajectories and derived phenotypes? These kinds of questions, and more, are facilitated by rigorous study of syndromes.

## CONFLICT OF INTEREST

The authors have no conflicts of interest to declare.

## AUTHOR CONTRIBUTIONS

**Miranda A. Sinnott‐Armstrong:** Conceptualization (equal); funding acquisition (equal); investigation (lead); visualization (equal); writing – original draft (equal); writing – review and editing (lead). **Rocio Deanna:** Conceptualization (supporting); investigation (supporting); writing – original draft (supporting); writing – review and editing (supporting). **Chelsea Pretz:** Conceptualization (equal); investigation (supporting); writing – original draft (supporting). **Jesse C. Harris:** Conceptualization (supporting); investigation (supporting); writing – original draft (supporting). **Amy Dunbar‐Wallis:** Conceptualization (supporting); investigation (supporting); writing – original draft (supporting). **Sukuan Liu:** Conceptualization (supporting); investigation (supporting); writing – original draft (supporting). **Stacey D. Smith:** Conceptualization (supporting); funding acquisition (equal); investigation (supporting); supervision (supporting); writing – review and editing (supporting). **Lucas C. Wheeler:** Conceptualization (equal); investigation (equal); supervision (lead); visualization (equal); writing – original draft (equal); writing – review and editing (equal).

## DATA AVAILABILITY STATEMENT

No data were generated in this work.
